# Complete genome sequences of *Chitinophaga* sp. strain MM2321 and *Microbacterium* sp. strain MM2322, isolated from a sand sample in Harsewinkel, Germany

**DOI:** 10.1128/mra.00700-24

**Published:** 2024-09-26

**Authors:** Marion Eisenhut, Tim Schulze, Fabian Niklas Jasper Matten, Annika Sengewald, Patrick Böhner, Daniel Falk, Carla Valeria Klinge, Ronja Langschwager, Stella Lohoff, Niklas Meyer, Vladyslava Ponomarchuk, Friederike Pünter, Jaclyn Sherlock, Diana Pschik, Levin Joe Klages, Tobias Busche, Lutz Wobbe, Andrea Bräutigam, Bianca Laker

**Affiliations:** 1 Computational Biology, Faculty of Biology, Bielefeld University, Bielefeld, Germany; 2 Center for Biotechnology (CeBiTec), Bielefeld University, Bielefeld, Germany; 3 Biology, Bielefeld University, Bielefeld, Germany; 4 Microbial Genomics and Biotechnology, Center for Biotechnology (CeBiTec), Bielefeld University, Bielefeld, Germany; 5 Medical School East Westphalia-Lippe, Bielefeld University, Bielefeld, Germany; University of Maryland School of Medicine, Baltimore, Maryland, USA

**Keywords:** microbial, genomes

## Abstract

We cultivated bacteria contained in a sandy soil sample, isolated DNA from a single bacterial colony, and assembled from genomic reads the full genome sequence of *Chitinophaga* and *Microbacterium* strains, termed MM2321 and MM2322. Besides the genome sequences, the phylogenetic classifications of both strains are reported.

## ANNOUNCEMENT

Soil is a rich habitat for a still underexplored plethora of organisms. Members of the genus *Chitinophaga* or *Microbacterium* are often isolated from soil samples. *Chitinophaga* are Gram-negative, facultatively anaerobic, rod-shaped bacteria (1). The genus *Microbacterium* contains Gram-positive, aerobic, rod-shaped bacteria (2). This study aims to provide additional genome sequences and thus improve the understanding of soil biodiversity.

**Fig 1:**
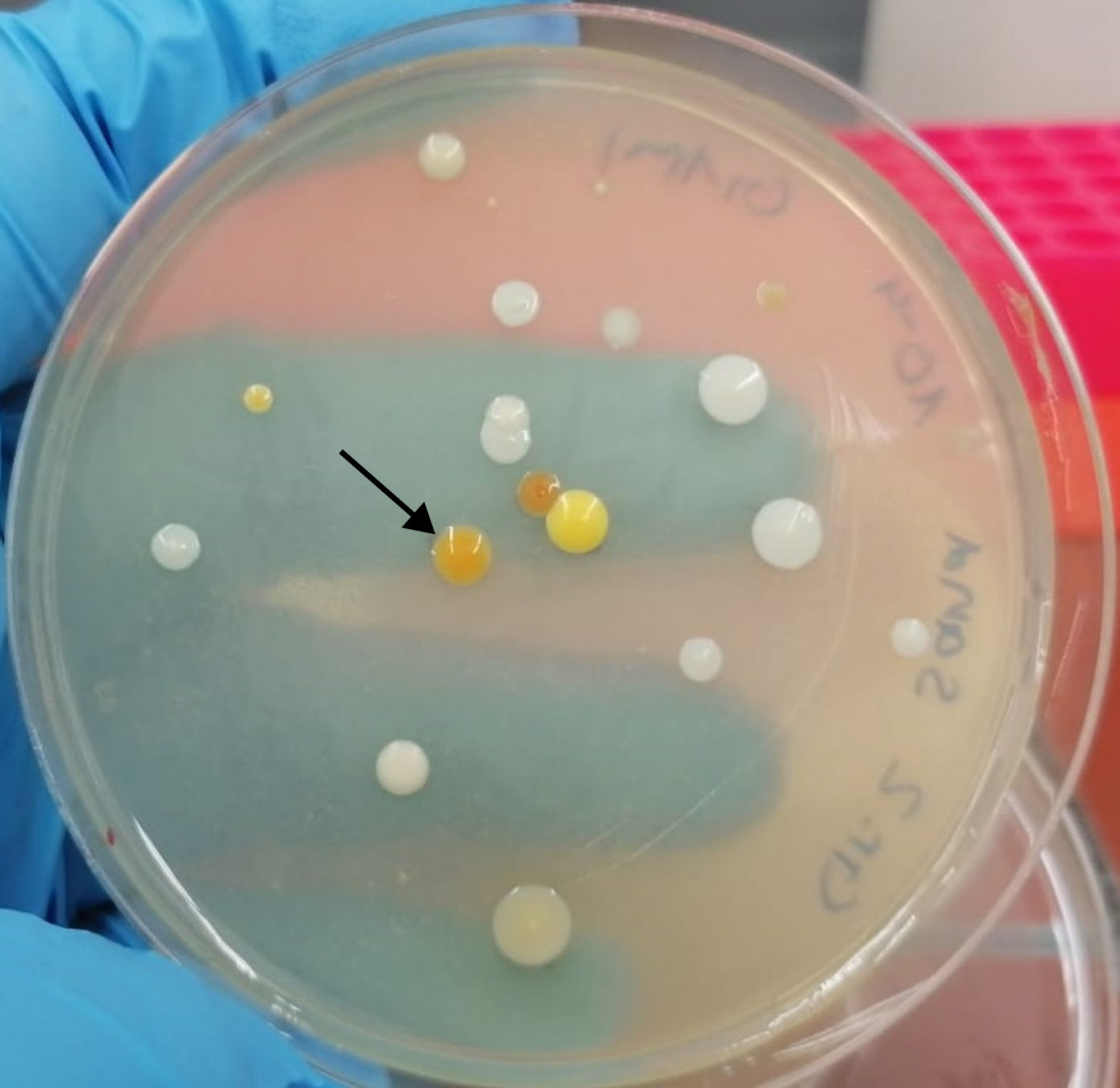
Bacteria isolated from the soil sample. A single orange-colored colony (labeled with black arrow) was picked for streaking on a fresh plate and subsequent DNA isolation.

A sandy soil sample from 5 cm below ground was collected at a forest near Harsewinkel, Germany (51°56'12.4"N; 8°15'15.0"E), suspended in 0.9% (wt/vol) NaCl, filtrated with a cellulose filter (pore size 4–12 µm), and centrifuged. The cell pellet was resuspended in fresh 0.9% NaCl. Dilutions were plated on agar medium [see (3)]. After incubation for 7 days at 30°C, a single orange-colored colony (Fig. 1) was picked and streaked on agar plates. After 48-hour incubation, DNA was isolated from the cell material obtained after streaking using the NucleoSpin Microbial DNA kit (Macherey-Nagel, Düren, Germany) with extra RNA digestion. Sequencing libraries were prepared using the ligation sequencing kit V14 (SQK-LSK114; Oxford Nanopore Technologies [ONT], Oxford, UK) following the manufacturer´s protocol. One R10.4.1 flow cell was run for 38 hours on a GridION (ONT). Bases were called using the super-accurate base-calling model of MinKNOW v22.07.9 (ONT), resulting in 200,456 raw reads with an *N_50_* of 12,725 bp. All programs were run with default parameters unless otherwise specified. Adapter trimming was performed with Porechop v0.2.4 (4). The expected genome size was estimated from a Web-BLAST against the NCBI’s standard databases (5). A genome assembly was calculated with Canu v2.2 (6) using a genome size of 8 Mbp. The assembled sequences were twice polished with racon v1.5.0 (7) and minimap v2.26 setting parameter “-ax map-ont” (8) and twice with Medaka v1.8.0 setting “-m r1041_e82_260bps_sup_g632” (ONT). Overlaps were trimmed with Berokka v0.2.3 (https://github.com/tseemann/berokka). Duplicated sequences were removed with the “clean” function of Circlator v1.5.5 (9), and low coverage sequences were removed (coverage <= 6.0). Sequences were oriented according to *dnaA* with the “fixstart” function. To determine the organism and strain type, the two resulting sequences were analyzed with TYGS (10) each. The ~6 Mbp large sequence was determined as *Chitinophaga* sp. and the ~3 Mbp large sequence as *Microbacterium* sp. Both sequences were separated for subsequent analyses. Genome completeness was investigated with BUSCO v5.5.0 setting “--mode geno” (11). Genes were predicted with Prokka v1.14.6 (12). Relevant statistics for the genome sequences and their genes are listed in Table 1.

**TABLE 1:** Sequencing and assembly statistics for *Chitinophaga* sp. strain MM2321 and *Microbacterium* sp. strain MM2322

	Finding for	
**Parameter**	***Chitinophaga* sp. strain MM2321**	***Microbacterium* sp. strain MM2322**
Genomic sequence		
Length (bp)	6,778,472	3,085,801
GC content (%)	45.09	69.22
Genome coverage (x)	29.69	32.27
Gene annotation		
Total no. of genes	5,461	3,003
No. of protein-coding genes	5,385	2,948
No. of rRNAs	15	6
No. of tRNAs	60	48
No. of transfer messenger RNAs	1	1
BUSCO results (%)*^a^*		
Complete	99.2	99.8
Single copy	98.5	99.6
Duplicated	0.7	0.2
Fragmented	0.5	0.2
Missing	0.3	0.0

^*a*^ The databases used (and the number of searched BUSCOs) were as follows: MM2321, bacteroidetes_odb10 (402 BUSCOs); MM2322, micrococcales_odb10 (537 BUSCOs).

The genomes of the here presented *Chitinophaga* sp. strain MM2321 and *Microbacterium* sp. strain MM2322 were both determined as complete since they were both assembled as circular sequences and the BUSCO value is above 99%. MM2321 has *Chitinophaga niastensis* DSM 24859 (GenBank accession number GCA_003014755.1) as the closest relative. A flexirubin gene cluster similar to that of *Chitinophaga pinensis* DSM 2588 (13) was identified and matches the yellow/orange appearance of the colonies (Fig. 1). MM2322 has *Microbacterium oleivorans* NBRC 103075 (GenBank accession number GCA_001552475.1) as the closest relative.

## Data Availability

The MM2321 and MM2322 assemblies, gene annotations, and reads are available in GenBank/ENA under BioProject accession number PRJEB73789. The GenBank/ENA accession number for the raw reads is ERS18417624. The GenBank/ENA accession number for the MM2321 assembly and annotation is GCA_964033635.1, and the number for the MM2322 assembly is GCA_964186585.1.
